# Herbal Formula Gegen-Qinlian Decoction for Type 2 Diabetes Mellitus: A Meta-Analysis of Randomized Controlled Trials

**DOI:** 10.1155/2020/3907920

**Published:** 2020-10-21

**Authors:** Lin Ren, Yanxia Cheng, Feng Qin

**Affiliations:** ^1^Department of Pharmacy, West China Hospital, Sichuan University, Chengdu 610041, China; ^2^Andrology Laboratory, West China Hospital, Sichuan University, Chengdu 610041, China

## Abstract

**Background:**

Herbal formula Gegen-Qinlian Decoction (GQD) has been widely used in China for the treatment of type 2 diabetes mellitus (T2DM), but its efficacy and safety are unclear.

**Method:**

The studies were identified from the PubMed, EMBASE, Cochrane Library, China National Knowledge Infrastructure database, Wanfang database, and VIP database using the keywords “Gegenqinlian” or “Gegen-Qinlian” or “Gegen-Qin-Lian” or “Ge Gen Qin Lian.” Relevant studies were selected according to predefined inclusion and exclusion criteria. Study selection, data extraction, and validation were carried out by, at least, two reviewers with disagreements being settled by discussion.

**Results:**

After literature search, a total of 26 randomized controlled trials were included with a total of 2553 patients. There was evidence that compared with metformin, the combination of GQD and metformin significantly reduced the fasting plasma glucose levels (MD −1.79, 95% CI (−2.31, −1.27), *p* < 0.00001); 2-hour postprandial plasma glucose levels (MD −1.72, 95% CI (−2.12, −1.31), *p* < 0.00001); and glycosylated hemoglobin levels (MD −1.26, 95% CI (−1.80, −0.72), *p* < 0.00001), and no serious side effects were identified.

**Conclusion:**

These data suggest that GQD may be an effective herbal formula in treating T2DM without serious side effects. The addition of GQD also enhances the hypoglycemic effects of metformin. However, the evidence remains weak due to methodological flaws, which may amplify the therapeutic benefit of GQD.

## 1. Introduction

Diabetes is a common and frequently occurring disease, seriously harming the human health. Diabetes is a group of clinical syndromes characterized by hyperglycemia. The main types are type 1 diabetes, type 2 diabetes, and gestational diabetes, and type 2 diabetes is the most prevalent form [1]. Type 2 diabetes mellitus (T2DM) is a metabolic disease characterized by chronic inflammation, insulin resistance, and islet cells damagement [2]. Long-term chronic hyperglycemia may cause microvascular disease, neuropathy, retinopathy, diabetic foot, diabetic nephropathy, and other diseases [3, 4]. Metformin is currently used as the first-line choice for the pharmacologic treatment of T2DM, but 20–30% of people develop gastrointestinal side effects, and 5% are unable to tolerate metformin due to these side effects [5]. Therefore, it has been gaining significant importance to search better agents worldwide from herbs or natural products in the recent years.

Gegen-Qinlian Decoction (GQD) is a classical herbal formula, which was firstly recorded in *Shang-Han-Lun* (Treatise on Febrile Diseases) of the *Han* Dynasty (202 BC-220 AD). GQD is widely used to treat diarrhea and diabetes in Chinese clinical practice [6–8]. It contains the following 4 herbs: Gegen (Puerariae Lobatae Radix), Huanglian (Coptidis Rhizoma), Huangqin (Scutellariae Radix), and Gancao (Glycyrrhizae Radix et Rhizoma). It had been reported that GQD could significantly decrease fasting blood glucose, glycosylated serum protein, glycosylated hemoglobin, and fasting serum insulin and promote myocardial glycolysis in diabetic rats [9, 10]. Isoflavonoids (3′-hydroxy puerarin, puerarin, daidzin, daidzein, genistin, and genistein), flavonoids (baicalin, baicalein, wogonoside, wogonin, liquiritin, and liquiritigenin), alkaloids (berberine, jatrorrhizine, palmatine, and coptisine), and glycyrrhetic acid have been identified within the preparation [11–13], and these components are correlated to the antidiabetic, antioxidant, and immunoregulative effects [14–17]. Notably, the administration of GQD has also yielded a potential hypoglycemic effect associated with multitarget therapy.

The greatest hindrance for the acceptance of herbal formula in the world is the scientific evaluation. Despite the extensive use of GQD in China, most of the evidence about GQD are anecdotal and have not been properly studied with scientifically rigorous trials, especially on human subjects.

The primary objective of this study is to determine the effectiveness and safety of GQD in the treatment of T2DM utilizing a meta-analysis approach. At the same time, we hope to find out the deficiencies in the use of GQD and find the direction for future research.

## 2. Methods

### 2.1. Literature Search

The study was registered in the PROSPERO database with ID CRD42020209404. No language restriction was imposed on the literature search. The literature search was performed using a combination of the term T2DM and the following keywords: Gegenqinlian or Gegen-Qinlian or Gegen-Qin-Lian or Ge-Gen-Qin-Lian. The databases that were searched included PUBMED (1966 to December 2019), EMBASE (1980 to December 2019), CNKI database (1994 to December 2019), Wanfang Data (1989 to December 2019), VIP Information (1990 to December 2019), and the Cochrane Library (Issue 12, 2019). A secondary search was also conducted by searching reference lists from primary studies, as well as former reviews.

### 2.2. Inclusion and Exclusion Criteria

Two reviewers (L.R. and F.Q.) independently decided which trials fit the inclusion and exclusion criteria for this study. Studies were eligible for inclusion if they met all of the following criteria: (1) study design: all participants were randomly allocated to an experimental group and a control group, and both parallel and crossover studies were eligible. (2) Target population: all participants were aged 18 years and above. (3) Diagnostic criteria: all participants were diagnosed as having T2DM according to the China guideline for T2DM [18] or WHO diagnostic criteria for T2DM [19]. (4) Comparison: studies had to compare GQD with metformin. (5) Outcome: studies have used data based on fasting plasma glucose (FPG) or 2 Hours Postprandial Plasma Glucose (2hPPG), or glycosylated hemoglobin (HbA1c) as primary outcomes.

Studies were eligible for exclusion if they met any of the following criteria: (1) case reports, animal studies, nonclinical studies, and reviews. (2) Unverified randomized controlled trial (RCT). (3) No appropriate experimental group or control group. (4) Duplicate publications.

### 2.3. Data Extraction

Two independent reviewers (L.R. and F.Q.) extracted data from the relevant studies using a standard data collection form in order to avoid bias in the process. All data were assessed for internal consistency, and inconsistencies were discussed by the three reviewers (L.R., F.Q., and Y.X.C.) when necessary. The following information was obtained: (1) the name of the author, (2) the date of publication, (3) the duration of the treatment, (4) the diagnostic criteria, (5) the age and gender of the participants, (6) the course of the disease, (7) the number of participants, (8) the intervention drugs (dosage and preparations), (9) primary outcomes, and (10) side effects. When necessary, additional information was collected through collaboration with the authors.

The quality assessment of the RCTs was also determined independently by two reviewers (L.R. and F.Q.) using the Cochrane risk of bias tool. Discrepancies were discussed by the three reviewers (L.R., F.Q., and Y.X.C.). According to our previous method [20], the study was designed to assess (1) random sequence generation, (2) allocation concealment, (3) blinding of participants and personnel, (4) blinding of outcome assessment, (5) reporting of dropout or withdrawal, (6) selective outcome reporting, and (7) other potential bias. Each item was rated as low risk of bias, high risk of bias, or unclear risk of bias.

### 2.4. Statistical Analysis

The trials that were included in the present study performed the following comparisons: GQD versus metformin; GQD plus metformin versus metformin. Meta-analysis was performed using Review Manager Software (version 5.4, Cochrane Collaboration and Updated Software). Mean difference (MD) with 95% confidence intervals (CI) was calculated since the data units were consistent. A fixed-effect model can be more appropriate when there is statistical homogeneity (*p* > 0.1 or an *I*^2^ statistic < 50%) among the studies, and random-effect model need to be pursued when statistical heterogeneity (*p* < 0.1 or an *I*^2^ statistic > 50%) exists in the trials. Funnel plot analysis and Egger's test were used to detect publication bias.

## 3. Results

### 3.1. Literature Search

**Figure 1:**
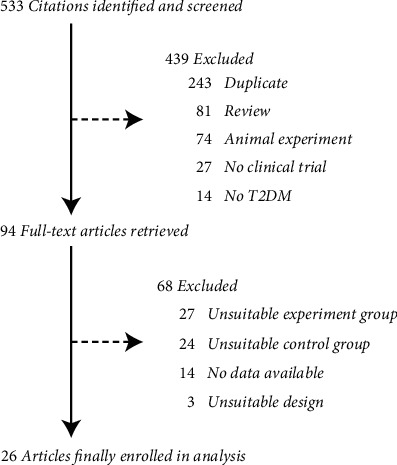
Study selection process for the meta-analysis.

An overview of the study selection process is summarized in Figure 1. The electronic search strategy identified 533 potentially relevant studies after accurate evaluation of the abstracts. In cases of disagreement as to whether an article was relevant, the full original article was retrieved for assessment. Of the 533 resulting studies, there were 243 duplicates, 81 reviews, 74 animal experiments, 27 nonclinical trials, and 14 not related to T2DM. After reading the full texts of the remaining 94 studies, 24 control groups were not metformin or unknown, 27 treatment groups contained other herbal medicine, 14 studies failed to provide useful data, 3 studies were not designed properly, and the remaining 26 studies were included in the meta-analysis [21–46].

### 3.2. Methodological Quality of Studies Included

**Figure 2:**
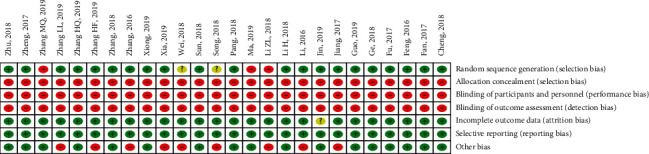
Methodological quality assessment of the risk of bias. Low risk of bias; unclear risk of bias; and high risk of bias.

According to the Cochrane risk of bias assessment tool, the methodologic quality item for all included studies is described in Figure 2. In general, the methodological quality of the 26 studies was low. Of the 26 studies, 21 studies reported random sequence generation, 3 studies have high risk on generating random sequence [29, 41, 46], and the other 2 studies did not describe random sequence generation [28, 31]. All the RCTs failed to describe the allocation concealment, blinding of participants and personnel, and blinding of outcome assessors in detail. There was no missing data in all the studies except the study of Jin [43], which did not mention the number of people who completed. The dose of metformin in 9 studies was not fixed, which was judged as high risks of other potential bias [26–29, 31, 32, 37, 38, 44].

### 3.3. Study Characteristics

Twenty-six studies, with 2553 participants, were included in the meta-analysis. All of the studies were performed in China. The studies were conducted between June 2010 and December 2019, and the dates of publication ranged from 2012 to 2019. The duration of intervention ranged from 2 to 24 weeks. Twenty-six trials reported data for FPG (*n* = 2553), 22 for 2hPPG (*n* = 2261), and 20 for HbA1c (*n* = 2006). GQD species and dose used varied between studies.  Table 1 summarizes the characteristics of each trial.

### 3.4. Pooled Effects of GQD on T2DM

#### 3.4.1. Improvement of Fasting Plasma Glucose

**Figure 3:**
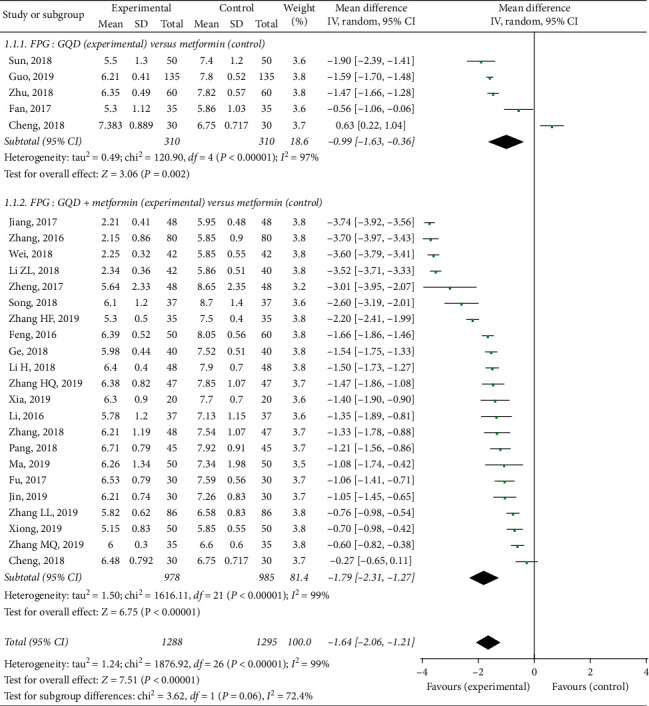
Treatment effects of GQD on FPG in patients with type 2 diabetes mellitus. Pooled estimates calculated by the random-effect method. FBG, fasting blood glucose; GQD, Gegen-Qinlian Decoction; CI, confidence interval; and IV, inverse variance.

Twenty-six randomized controlled trials tested the effect of GQD on FPG in patients with T2DM [21–46]. A high level of statistical heterogeneity was observed for the meta-analysis of FBG (*I*^2^ = 99%, *p* < 0.00001), so the random-effect model was used. As presented in Figure 3, the meta-analysis identified a significant decrease of the FPG compared to control group (MD −1.64, 95% CI (−2.06, −1.21), *p* < 0.00001). Similar results were reported in the subgroup analysis, GQD had a significantly lower FPG than the only metformin group (MD −0.99, 95% CI (−1.63, −0.36), *p*=0.002; and GQD plus metformin had a significantly lower FPG than the only metformin group (MD −1.79, 95% CI (−2.31, −1.27), *p* < 0.00001).

#### 3.4.2. Improvement of 2-Hours Postprandial Plasma Glucose

**Figure 4:**
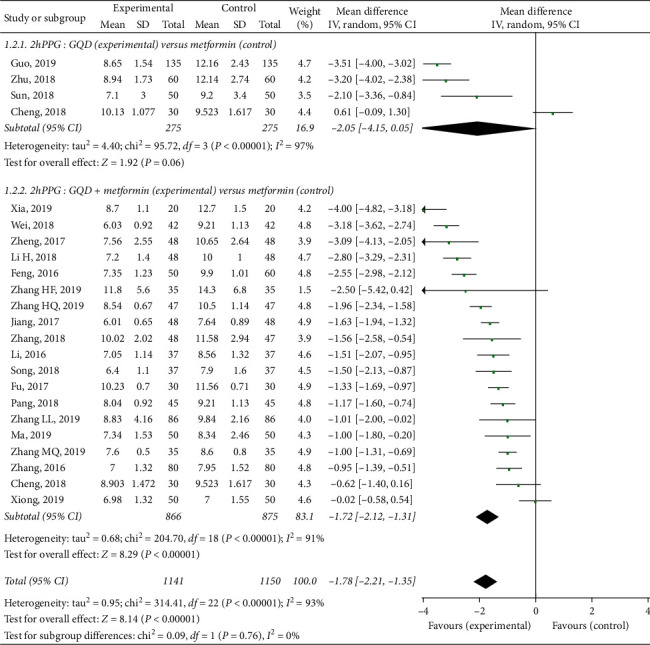
Treatment effects of GQD on 2hPPG in patients with type 2 diabetes mellitus. Pooled estimates calculated by the random-effect method. 2hPPG, 2-hour postprandial plasma glucose; GQD, Gegen-Qinlian Decoction; CI, confidence interval; and IV, inverse variance.

Twenty-two randomized controlled trials tested the effect of GQD on 2hPPG in patients with T2DM [21–23, 25–28, 30–33, 35–42, 44–46]. A high level of statistical heterogeneity was observed for the meta-analysis of 2hPPG (*I*^2^ = 93%, *p* < 0.00001), so the random-effect model was used. As presented in Figure 4, results of this meta-analysis showed that GQD could not significantly reduce 2hPPG, as compared to metformin (MD −2.05, 95% CI (−4.15, 0.05), *p*=0.06. However, GQD plus metformin had a significantly lower 2hPPG than the only metformin group (MD −1.72, 95% CI (−2.12, −1.31), *p* < 0.00001).

#### 3.4.3. Improvement of Glycosylated Hemoglobin

**Figure 5:**
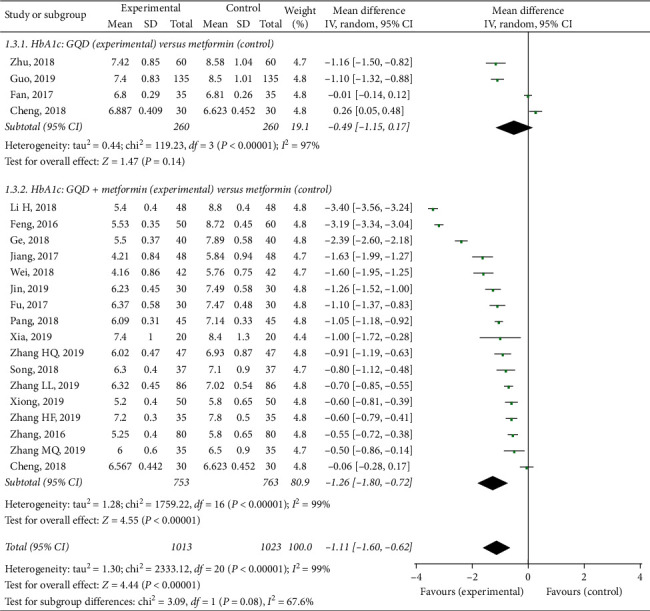
Treatment effects of GQD on HbA1c in patients with type 2 diabetes mellitus. Pooled estimates calculated by the random-effect method. HbA1c, Glycosylated hemoglobin; GQD, Gegen-Qinlian Decoction; CI, confidence interval; and IV, inverse variance.

Twenty randomized controlled trials tested the effect of GQD on HbA1c in patients with T2DM [22–28, 31–37, 40, 42–46]. A high level of statistical heterogeneity was observed for the meta-analysis of HbA1c (*I*^2^ = 99%, *p* < 0.00001), so the random-effect model was used. As presented in Figure 5, results of this meta-analysis showed that GQD could not significantly reduce HbA1c, as compared to metformin (MD −0.49, 95% CI (−1.15, 0.17), *p*=0.14). However, GQD plus metformin had a significantly lower HbA1c than the only metformin group (MD −1.26, 95% CI (−1.80, −0.72), *p* < 0.00001).

### 3.5. Publication Bias

**Figure 6:**
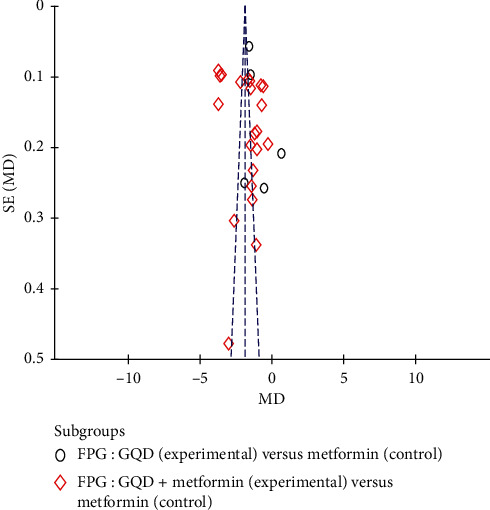
Funnel plots of randomized controlled trials of GQD. GQD, Gegen-Qinlian Decoction; FBG, fasting blood glucose.

The prevention of publication bias is important for the scientific perspective. In this study, the funnel plots showed that no evidence of publication bias was apparent in the 26 clinical trials Figure 6, and Egger's test also indicated no significant publication bias (*p*=0.2470).

## 4. Adverse Events

Eleven RCTs reported information on adverse effects [21–25, 34, 36, 41, 43, 44, 46]. There were no serious adverse reactions in the RCTs, mostly mild to moderate gastrointestinal reactions. In these RCTs, the most common adverse events were nausea, vomiting, diarrhea, headache, and hypoglycemia. The adverse events of GQD were nausea, vomiting, diarrhea, and hypoglycemia, which is similar to the side effects of metformin [23, 24]. However, the adverse events in the combination of GQD and metformin were decreased significantly in three studies [41, 43, 46], as compared to the metformin group.

## 5. Discussion

In total, this study assessed the efficacy and safety of GQD in adult patients with T2DM. Review Manager 5.4 software was used to analyze the clinical data from 26 RCTs, with a total of 2553 participants. All trials were carried out in China, and all the patients involved were Chinese. The results showed that the combination of GQD and metformin was more effective in the treatment of T2DM when compared to metformin alone. This study also suggested that GQD was a safe drug for T2DM patients.

Diabetes is an increasingly important condition in the world. In 2011, there are 366 million patients with diabetes, and it is expected to rise to 552 million by 2030 [47]. T2DM is part of a complex metabolic-cardiovascular syndrome, and metformin is recommended as first-line oral therapy in most national and international guidelines. However, a considerable number of patients need to add other drugs on the basis of first-line metformin over time [48]. Traditional Chinese medicine advocates the use of multiple herbal medicines in combination, which can not only produce multiple effects but also reduce adverse reactions [49]. In the recent years, GQD has played an important role in the treatment of T2DM in China [9, 10]. Many studies have found that the chemical components of GQD are related to the pathological factors of T2DM. Puerarin, the component of Puerariae Lobatae Radix, plays a role in reducing blood sugar by promoting insulin expression and improving glucose metabolism [16]. The flavonoids of Puerariae Lobatae Radix not only have a significant hypoglycemic effect but also can prevent the diabetic complications [50]. Berberine, the component of Coptidis Rhizoma, has significant effects on reducing blood glucose and blood lipid, improving insulin resistance and egulating intestinal tract flora [51]. Baicalin, the main active ingredient of Scutellariae Radix, can promote glucose uptake and glycolysis, inhibit gluconeogenesis, and improve glucose metabolism [52]. Isoliquiritigenin and liquiritigenin, the components of Glycyrrhizae Radix et Rhizoma, can reduce insulin resistance in liver [53]. It is noteworthy that the active ingredients of GQD have produced a potential effect of multitarget therapy. The explanation might be that a disease, such as T2DM, with most likely a number of various mechanisms involved, is likely to respond to a multitarget treatment.

Metformin can reduce glucose production and partially increased glucose utilization, while GQD can accelerate the absorption and utilization [5, 9, 10, 48, 50–54]. These findings suggest that the combined use of GQD and metformin may be more effective than metformin alone. Indeed, as shown in Figures 3–5, the present study included 26 RCTs involving 2553 patients. The results showed that compared with metformin, GQD plus metformin significantly improve FPG (MD −1.64, *p* < 0.00001), 2hPPG (MD −1.78, *p* < 0.00001), and also significantly improve the HbA1c (MD −1.11, *p* < 0.00001). Additionally, the GQD plus metformin showed a significant reduction in adverse events when compared with the patients without GQD treatment.

In 2017, the study of Ryuk et al. [7] confirmed the synergistic effect of GQD plus metformin on glucose control, which included 5 RCTs with a total of 499 participants. In our study, which is based on the other three indicators (FPG, 2hPPG, and HbA1c), we further determine the effectiveness and safety of GQD in the treatment of T2DM. FPG is an indicator for the diagnosis of diabetes. 2hPPG provides more information on postprandial glycemic control and is important in prognostic indicators such as cardiovascular disease, renal failure, or diabetic amputation. HbA1c provides information on overtime blood glucose control [55]. In addition, more RCTs (26 studies included) and more participants (more than 2500 patients) were included in this study, and the results further confirmed that the combination of GQD and metformin was more effective compared to metformin alone in the treatment of T2DM with no serious side effects.

The present meta-analysis has several potential limitations that should be addressed. First, the therapeutic effect of herbal formula has been gradually recognized by international medical community. But, all RCTs included are conducted in China and published in Chinese, which has seriously affected the international communication of GQD. Secondly, traditional Chinese medicine has always emphasized individualized treatment based on clinical symptoms, so different medicines, different doses, and different courses of treatment may lead to heterogeneity. The high heterogeneity is observed among included RCTs, which will influence the analysis, interpretation, and conclusions of this study. Third, quality control of herbal formula has been necessary and urgent for the safety and efficacy of GQD, but all the RCTs lack sufficient information on the quality control. Fourth, the traditional decoction is influenced by many factors, such as the quality of medicinal materials and the method of decocting and taking. In the recent years, some new forms of herbal formula (such as capsules or tablets) have been studied and popularized, which helps GDQ to be safer and more stable in quality and curative effect [56, 57]. Finally, as shown in Figure 2, lacking of detailed demographic and methodological information in many studies (such as medication history, sequence generation, and dropout rates) leads to the poor methodological quality. Despite the limitations, this study confirms that GQD is indeed a safe and effective adjunct to metformin for the treatment of T2DM, and the consistent and highly significant are very compelling.

## 6. Conclusions

This study could not provide adequate evidence to conclude whether GQD is superior, inferior, or the same as metformin in terms of efficacy for the treatment of T2DM. However, the hypoglycemic effect of metformin is significantly enhanced when it is combined with GQD, and no serious side effects are identified. Due to overall limited quality of included studies, the therapeutic benefit of GQD can be substantiated to a limited degree. Future studies are needed to address the effectiveness and safety of GQD with larger sample size and better methodological quality across diverse populations.

## Figures and Tables

**Table 1:** Characteristics of the included studies in meta-analysis.

Study	Diagnostic criteria	Number of participants	Male proportion	Age (years)	Course of T2DM (years)	Intervention drugs (dosage)	Treatment course	Outcome	Adverse event
Sun, 2018 [21]	WHOT2D	A: 50	A: 56.0%	A: 56.8 ± 6.5	A: 2.6 ± 0.5	A: GQD (30.5 g, BID)	8 weeks	FPG, 2hPPG	Included
		B: 50	B: 52.0%	B: 55.8 ± 5.6	B: 2.9 ± 0.8	B: metformin (250 mg, BID)			

Guo, 2019 [22]	CGT2D	A: 135	A: 51.8%	A: 60.5 ± 1.4	A: 6.6 ± 1.7	A: GQD (60 g, BID)	24 weeks	FPG, 2hPPG, HbA1c	Included
		B: 135	B: 52.6%	B: 60.4 ± 1.3	B: 6.5 ± 1.6	B: metformin (<2000 mg/d)			

Zhu, 2018 [23]	CGT2D	A: 60	A: 56.7%	A: 52.3 ± 6.8	A: 6.7 ± 3.2	A: GQD (60 g, BID)	24 weeks	FPG, 2hPPG, HbA1c	Included
		B: 60	B: 55.0%	B: 52.0 ± 6.8	B: 6.7 ± 3.1	B: metformin (250–500 mg, TID)			

Fan et al., 2017 [24]	WHOT2D	A: 35	A: 60.0%	A: 36.4 ± 7.1	A: 3.1 ± 1.7	A: GQD (30.5 g, BID)	8 weeks	FPG, HbA1c	Included
		B: 35	B: 54.3%	B: 38.0 ± 6.5	B: 3.4 ± 1.5	B: metformin (850 mg, BID)			

Cheng, 2018 [25]	CGT2D	A: 30	A: 46.7%	A: 54.4 ± 6.5	NR	A: GQD (26.5 g, BID)	4 weeks	FPG, 2hPPG, HbA1c	Included
		C: 30	C: 43.3%	C: 54.2 ± 6.0		C: GQD (26.5 g, BID) + metformin (500 mg, TID)			
		B: 30	B: 56.7%	B: 55.9 ± 6.0		B: metformin (500 mg, TID)			

Jiang, 2017 [26]	WHOT2D	C: 48	C: 54.2%	C: 48.9 ± 3.1	C: 5.0 ± 1.0	C: GQD (25 g, BID) + metformin (0.25–1 g, BID)	12 weeks	FPG, 2hPPG, HbA1c	NR
		B: 48	B: 58.3%	B: 48.5 ± 3.2	B: 4.8 ± 0.8	B: metformin (0.25–1 g, BID)			

Zhang and Cai, 2016 [27]	WHOT2D	C: 80	C: 56.2%	C: 44.5 ± 11.3	NR	C: GQD (60 g, BID) + metformin (0.25–1 g, BID)	8 weeks	FPG, 2hPPG, HbA1c	NR
		B: 80	B: 58.8%	B: 43.8 ± 10.5		B: metformin (0.25–1 g, BID)			

Wei, 2018 [28]	WHOT2D	C: 42	C: 61.9%	C: 47.5 ± 6.0	C: 4.5 ± 2.4	C: GQD (NR, BID) + metformin (NR)	12 weeks	FPG, 2hPPG, HbA1c	NR
		B: 42	B: 57.1%	B: 47.2 ± 5.1	B: 4.1 ± 2.3	B: metformin (NR)			

Li, 2018 [29]	WHOT2D	C: 40	NR	C: 52.1 ± 4.2	C: 6.8 ± 3.4	C: GQD (60 g, BID) + metformin (0.25–1 g, BID)	8 weeks	FPG	NR
		B: 42		B: 51.7 ± 3.9	B: 6.3 ± 2.5	B: metformin (0.25–1 g, BID)			

Zheng, 2017 [30]	WHOT2D	C: 48	C: 58.3%	C: 52.3 ± 8.3	C: 5.7 ± 1.2	C: GQD (60 g, BID) + metformin (500 mg, BID)	8 weeks	FPG, 2hPPG	NR
		B: 48	B: 56.2%	B: 53.5 ± 8.3	A: 5.6 ± 1.4	B: metformin (500 mg, BID)			

Song, 2018 [31]	WHOT2D	C: 37	C: 54.0%	C: 61.2 ± 3.1	C: 3.1 ± 0.3	C: GQD (BID) + metformin (500–2000 mg/d)	4 weeks	FPG, 2hPPG, HbA1c	NR
		B: 37	B: 56.8%	B: 60.2 ± 3.2	B: 3.3 ± 0.2	B: metformin (500–2000 mg/d)			

Zhang et al., 2019 [32]	WHOT2D	C: 35	NR	C: 35–70	NR	C: GQD (25 g, BID) + metformin (≤2 g/d)	8 weeks	FPG, 2hPPG, HbA1c	NR
		B: 35		B: 36–71		B: metformin (≤2 g/d)			

Feng et al., 2016 [33]	WHOT2D	C: 50	60.0%	C: 57.2 ± 9.8	C: 1.0 ± 0.2	C: GQD (23.5 g, BID) + metformin (500 mg, QD)	12 weeks	FPG, 2hPPG, HbA1c	NR
		B: 50		B: 56.5 ± 10.3	B: 1.0 ± 0.1	B: metformin (500 mg, QD)			

Ge, 2018 [34]	CGT2D	C: 40	C: 62.5%	C: 50.6 ± 7.2	C: 1.1 ± 0.3	C: GQD (23.5 g, BID) + metformin (500 mg, QD)	8 weeks	FPG, HbA1c	Included
		B: 40	B: 65.0%	B: 50.7 ± 7.3	B: 1.1 ± 0.2	B: metformin (500 mg, QD)			

Li, 2018 [35]	CGT2D	C: 48	C: 58.3%	C: 58.1 ± 9.7	C: 3.7 ± 1.5	C: GQD (23.5 g, BID) + metformin (500 mg, QD)	2 weeks	FPG, 2hPPG, HbA1c	NR
		B: 48	B: 54.2%	B: 59.4 ± 9.8	B: 3.5 ± 1.8	B: metformin (500 mg, QD)			

Zhang, 2019 [36]	CGT2D	C: 47	C: 53.2%	C: 45.5 ± 5.3	C: 5.4 ± 2.3	C: GQD (43 g, BID) + metformin (500 mg, TID)	8 weeks	FPG, 2hPPG, HbA1c	Included
		B: 47	B: 51.1%	B: 46.6 ± 5.5	B: 5.7 ± 2.4	B: metformin (500 mg, TID)			

Xia, 2019 [37]	CGT2D	C: 20	C: 60.0%	C: 58.2 ± 4.0	NR	C: GQD (60 g, BID) + metformin (0.25–0.5 g, TID)	24 weeks	FPG, 2hPPG, HbA1c	NR
		B: 20	B: 55.0%	B: 56.5 ± 3.8		B: metformin (0.25–0.5 g, TID)			

Li, 2016 [38]	WHOT2D	C: 37	C: 54.0%	50.3 ± 5.7	5.2 ± 1.2	C: GQD (31 g, BID) + metformin (500–2500 mg/d)	8 weeks	FPG, 2hPPG	NR
		B: 37	B: 56.8%			B: metformin (500–2500 mg/d)			

Zhang et al., 2018 [39]	WHOT2D	C: 48	C: 54.2%	C: 51.3 ± 6.8	C: 5.4 ± 2.3	C: GQD (39.5 g, BID) + metformin (500 mg, QD)	8 weeks	FPG, 2hPPG	NR
		B: 47	B: 53.2%	B: 51.2 ± 7.3	B: 5.6 ± 2.1	B: metformin (500 mg, QD)			

Pang et al., 2018 [40]	WHOT2D	C: 45	C: 51.1%	C: 53.5 ± 8.2	C: 5.5 ± 1.3	C: GQD (60 g, BID) + metformin (250 mg, TID)	8 weeks	FPG, 2hPPG, HbA1c	NR
		B: 45	B: 53.3%	B: 54.1 ± 8.3	B: 5.4 ± 1.1	B: metformin (250 mg, TID)			

Ma, 2019 [41]	WHOT2D	C: 50	C: 54.0%	C: 49.7 ± 2.6	C: 6.2 ± 2.9	C: GQD (31 g, BID) + metformin (500 mg, BID)	8 weeks	FPG, 2hPPG	Included
		B: 50	B: 56.0%	B: 50.5 ± 1.7	B: 6.6 ± 2.6	B: metformin (500 mg, BID)			

Fu, 2017 [42]	CGT2D	C: 30	C: 56.7%	C: 56.1 ± 8.2	C: 5.2 ± 2.1	C: GQD (53 g, BID) + metformin (500 mg, TID)	12 weeks	FPG, 2hPPG, HbA1c	NR
		B: 30	B: 53.3%	B: 57.5 ± 8.2	B: 5.6 ± 2.0	B: metformin (500 mg, TID)			

Jin et al., 2019 [43]	WHOT2D	C: 30	C: 53.3%	C: 58.1 ± 3.1	3.5 ± 0.9	C: GQD (30.5 g, BID) + metformin (500 mg, QD)	8 weeks	FPG, HbA1c	Included
		B: 30	B: 60.0%	B: 58.0 ± 3.7		B: metformin (500 mg, QD)			

Zhang et al., 2019 [44]	CGT2D	C: 86	C: 59.3%	C: 48.3 ± 10.9	NR	C: GQD (32.5 g, BID) + metformin (500–1500 mg/d)	8 weeks	FPG, 2hPPG, HbA1c	Included
		B: 86	B: 61.6%	B: 48.7 ± 11.1		B: metformin (500–1500 mg/d)			

Xiong, 2019 [45]	CGT2D	C: 50	C: 58.0%	C: 53.6 ± 7.6	C: 4.8 ± 1.0	C: GQD (60 g, BID) + metformin (250 mg, TID)	8 weeks	FPG, 2hPPG, HbA1c	NR
		B: 50	B: 60.0%	B: 53.5 ± 7.8	B: 4.8 ± 1.1	B: metformin (250 mg, TID)			

Zhang, 2019 [46]	CGT2D	C: 35	51.4%	C: 48.9 ± 3.4	C: 5.0 ± 0.5	C: GQD (25 g, BID) + metformin (500 mg, QD)	8 weeks	FPG, 2hPPG, HbA1c	Included
		B: 35		B: 48.5 ± 3.6	B: 4.9 ± 0.7	B: metformin (500 mg, QD)			

GQD, Gegen-Qinlian Decoction; T2DM, type 2 diabetes mellitus; A, GQD group; B, metformin group; C, GQD + metformin group; CGT2D, China guideline for T2DM; WHOT2D, World Health Organization guideline for T2DM; FPG, fasting plasma glucose; 2hPPG, 2-hours postprandial plasma glucose; HbA1c, glycosylated hemoglobin; QD, once a day; BID, twice a day; TID, three times a day; NR, no record.

## Data Availability

Data that support the results of this study are included within the article.
